# Using Smart Speakers in Low-Income Senior Housing to Enhance the Aging in Place Experience: Stakeholder Views

**DOI:** 10.1093/geroni/igab046.2494

**Published:** 2021-12-17

**Authors:** Jane Chung, Jodi Winship, Katherine Falls, Pamela Parsons, Michael Bleich

**Affiliations:** 1 Virginia Commonwealth University, Virginia, United States; 2 Virginia Commonwealth University, Richmond, Virginia, United States

## Abstract

Smart speakers provide a platform that can integrate smart home technology and/or safety devices within the home to enhance quality of life and independent living for older adults. However, few attempts to utilize this technology specifically within low-income senior housing (LISH) residents have been documented. Our purpose was to explore different stakeholder perceptions about the use of smart speakers to support aging in place in older adults living alone in LISH. Smart speakers were deployed in individual LISH apartments, equipped with a voice technology-based aging in place solution for the facility. A qualitative analysis of semi-structured interviews using a constant comparative approach for emerging themes was conducted (n=10: older adult users, n=2: housing staff, n=2: voice technology developers). The three participant groups showed diverging perceptions in terms of benefits, uses, and stakeholder interests. Older adults found smart speakers useful in four main areas: assistance with daily tasks, feeling connected, safety measures, and emotional wellbeing. The two other groups showed a broader interest in the use of the smart speaker device, such as residential management tools and communication channels in addition to its potential use as safety and wellness tools. Older adults experienced significant difficulty setting up desired functions or finding instructions, which restricted utilization of the technology to a limited set of tasks. All stakeholder groups addressed a need for formal training or personalized tech support for older adult users. Findings indicate the importance of developing deployment strategies tailored to the needs and characteristics of the target user group.

